# Positive Allosteric Modulation of CB_1_ Cannabinoid Receptor Signaling Enhances Morphine Antinociception and Attenuates Morphine Tolerance Without Enhancing Morphine- Induced Dependence or Reward

**DOI:** 10.3389/fnmol.2020.00054

**Published:** 2020-04-28

**Authors:** Richard A. Slivicki, Vishakh Iyer, Sonali S. Mali, Sumanta Garai, Ganesh A. Thakur, Jonathon D. Crystal, Andrea G. Hohmann

**Affiliations:** ^1^Program in Neuroscience, Indiana University, Bloomington, IN, United States; ^2^Department of Psychological and Brain Sciences, Indiana University, Bloomington, IN, United States; ^3^Center for Drug Discovery, Bouve College of Health Sciences, Northeastern University, Boston, MA, United States; ^4^Gill Center for Biomolecular Science, Indiana University, Bloomington, IN, United States

**Keywords:** endocannabinoid, allosteric modulator, neuropathic pain, opioid, withdrawal, isobologram

## Abstract

Opioid analgesics represent a critical treatment for chronic pain in the analgesic ladder of the World Health Organization. However, their use can result in a number of unwanted side-effects including incomplete efficacy, constipation, physical dependence, and overdose liability. Cannabinoids enhance the pain-relieving effects of opioids in preclinical studies and dampen unwanted side-effects resulting from excessive opioid intake. We recently reported that a CB_1_ positive allosteric modulator (PAM) exhibits antinociceptive efficacy in models of pathological pain and lacks the adverse side effects of direct CB_1_ receptor activation. In the present study, we evaluated whether a CB_1_ PAM would enhance morphine’s therapeutic efficacy in an animal model of chemotherapy-induced neuropathic pain and characterized its impact on unwanted side-effects associated with chronic opioid administration. In paclitaxel-treated mice, both the CB_1_ PAM GAT211 and the opioid analgesic morphine reduced paclitaxel-induced behavioral hypersensitivities to mechanical and cold stimulation in a dose-dependent manner. Isobolographic analysis revealed that combinations of GAT211 and morphine resulted in anti-allodynic synergism. In paclitaxel-treated mice, a sub-threshold dose of GAT211 prevented the development of tolerance to the anti-allodynic effects of morphine over 20 days of once daily dosing. However, GAT211 did not reliably alter somatic withdrawal signs (i.e., jumps, paw tremors) in morphine-dependent neuropathic mice challenged with naloxone. In otherwise naïve mice, GAT211 also prolonged antinociceptive efficacy of morphine in the tail-flick test and reduced the overall right-ward shift in the ED_50_ for morphine to produce antinociception in the tail-flick test, consistent with attenuation of morphine tolerance. Pretreatment with GAT211 did not alter somatic signs of μ opioid receptor dependence in mice rendered dependent upon morphine via subcutaneous implantation of a morphine pellet. Moreover, GAT211 did not reliably alter μ-opioid receptor-mediated reward as measured by conditioned place preference to morphine. Our results suggest that a CB_1_ PAM may be beneficial in enhancing and prolonging the therapeutic properties of opioids while potentially sparing unwanted side-effects (e.g., tolerance) that occur with repeated opioid treatment.

## Introduction

An estimated 11.2% of individuals in the United States are diagnosed with some form of chronic pain, representing an unmet clinical need for analgesics that are safe, effective and lack abuse liability (Jamison and Mao, 2015). Opioid-based therapies are effective tools for pain management and remain a component of the analgesic ladder of the World Health Organization (Ballantyne et al., 2016). However, these therapies are plagued by a myriad of unwanted side effects including constipation, nausea, tolerance, dependence and, in extreme cases, overdose death (Gardell et al., 2006; Fields, 2011; Roques et al., 2012; Jamison and Mao, 2015). These observations highlight an urgent need to develop alternative therapies that retain or increase the beneficial efficacy of currently prescribed analgesics while reducing their detrimental side effects.

Smoked or vaporized cannabis and cannabinoid-based extracts show analgesic efficacy in a number of different chronic pain populations (Hill, 2015; Hill et al., 2017). Cannabinoids exert their antinociceptive effects through activation of cannabinoid receptors, such as CB_1_ receptors, which are densely and heterogeneously expressed throughout the central nervous system (CNS) (Lu and Mackie, 2016). Direct activation of CB_1_ receptors produces antinociception in a number of different preclinical pain models (Woodhams et al., 2017). However, like opioid-based pharmacological approaches, a number of adverse side-effects accompany direct CB_1_ receptor activation. These on-target side effects include locomotor impairment, tolerance, and dependence (Woodhams et al., 2017). Thus, a number of drug discovery efforts have focused on indirectly activating CB_1_ receptors as a way to circumvent these unwanted side effects.

Allosteric modulation is a topic of substantial research interest for leveraging the therapeutic efficacy of commonly targeted G-protein coupled receptors such as μ opioid and CB_1_ receptors (Burford et al., 2013, 2015; Nguyen et al., 2016; Alaverdashvili and Laprairie, 2018; Dopart et al., 2018). Allosteric modulators bind to an allosteric binding site that is distinct from the orthosteric binding site that binds both the endogenous ligand and classical orthosteric agonists. Allosteric modulators produce conformational changes to the orthosteric binding site that can enhance (i.e., in the case of positive allosteric modulators) or negate (i.e., in the case of negative allosteric modulators) the affinity for orthosteric binding and/or receptor efficacy observed downstream of binding by an orthosteric ligand. In 2005, an allosteric binding site was first described on the CB_1_ receptor (Price et al., 2005). The identification of this allosteric binding site fostered drug discovery efforts that led to the generation of a number of positive allosteric modulators (PAMs) in efforts to elicit therapeutic effects of CB_1_ receptor activation while circumventing unwanted side-effects (Laprairie et al., 2017). Theoretically, an allosteric modulator affords better temporal and spatial resolution as it is only efficacious if the orthosteric site is occupied at the same time (Wootten et al., 2012), in contrast to an exogenous agonist which can activate the receptor independent of the presence of the endogenous ligand. Work from our laboratory and others suggest that CB_1_ PAMs show anti-allodynic efficacy in preclinical models of ocular, inflammatory and neuropathic pain without producing the characteristic cannabimimetic side-effect profile (Ignatowska-Jankowska et al., 2015; Cairns et al., 2017; Slivicki et al., 2018b; Garai et al., 2020; Thapa et al., 2020). We demonstrated that the CB_1_ PAM GAT211 suppressed inflammatory pain produced by intraplantar injection of complete Freund’s adjuvant as well as chemotherapy-induced neuropathic pain produced by paclitaxel without producing tolerance (Slivicki et al., 2018b). Moreover, GAT211 lacked cardinal signs of CB_1_ activation (i.e., immobility in the ring test, motor ataxia in the rota-rod test, tail-flick antinociception and hypothermia) following either acute or repeated dosing (Slivicki et al., 2018b). Thus, CB_1_ PAMs may represent a clinically valid alternative approach to exploit CB_1_-mediated antinociceptive efficacy without the unwanted side effects of direct acting CB_1_ agonists (Ignatowska-Jankowska et al., 2015; Slivicki et al., 2018b).

The endocannabinoid system, which includes cannabinoid receptors, their endogenous ligands and their respective hydrolytic and synthetic enzymes, has been implicated in opioid antinociceptive efficacy, tolerance, dependence and withdrawal (Yamaguchi et al., 2001; Cichewicz and Welch, 2003; Scavone et al., 2013). Effects of direct CB_1_ receptor agonists and endocannabinoid deactivation inhibitors on these measures are previously characterized (Yamaguchi et al., 2001; Cichewicz and Welch, 2003; Scavone et al., 2013). However, the impact of CB_1_ PAMs on these same traits remains unexplored.

Opioids remain widely used clinically for pain management. In the present study, we used a mouse model of chemotherapy-induced neuropathic pain induced by the taxane chemotherapeutic agent paclitaxel to evaluate the potential of a CB_1_ PAM (GAT211) (Laprairie et al., 2019) to enhance morphine’s anti-allodynic efficacy. Using isobolographic analysis, we asked whether the anti-allodynic effects of a CB_1_ PAM would synergize with the narcotic analgesic morphine. Because opioids also exhibit unwanted side-effects, including reward, tolerance and physical dependence, we evaluated whether therapeutic doses of the CB_1_ PAM would alter these unwanted side-effects of opioids. Our studies add to an emerging literature that characterizes effects of CB_1_ agonists and endocannabinoid tone modulators within the context of opioid antinociceptive tolerance and physical dependence. These questions are of importance when considering potential benefits and/or side effects associated with polydrug therapies for the treatment of pain and other disorders.

## Materials and Methods

### Animals

Adult male C57BL/6J mice (∼12 weeks of age, Jackson Laboratories) were used for all studies, except where noted. ICR mice (∼10 weeks of age, Envigo) were used for the morphine pellet studies as C57BL/6J exhibit higher mortality rates compared to ICR mice following subcutaneous implantation with 75 mg morphine pellets (Ramesh, 2012). Mice were single housed beginning 24 h prior to any treatment condition. Mice were maintained on a 12-h light/dark cycle (lights on from 7 AM to 7 PM) in temperature and humidity-controlled facility and allowed *ad libitum* access to food and water throughout the experimental period. All procedures were approved by the Bloomington Institutional Animal Care and Use Committee and followed guidelines outlined by the International Association for the Study of Pain (Zimmermann, 1983).

### Materials

Paclitaxel (Tecoland Corporation, Edison, NJ, United States) was dissolved in a vehicle consisting of 95% ethanol: cremophor: 0.9% saline in a 1:1:18 ratio and injected via the intraperitoneal (i.p.) route in a volume of 6.67 mL/kg. For pharmacological manipulations, morphine (National Institute on Drug Abuse, Bethesda, MD, United States), naloxone (Sigma Aldrich, St. Louis, MO, United States), and GAT211 (synthesized by the authors SG and GAT) were used. These compounds were dissolved in a vehicle consisting of 20% DMSO with the remaining 80% consisting of 95% ethanol: emulphor: saline in a 1:1:8 ratio and injected (i.p.) in a volume of 5 mL/kg. Doses of morphine used in assessments of tail-flick antinociception were dissolved in saline. Combination doses of GAT211 + morphine were co-administered so that overall injection volumes did not exceed 5 mL/kg. In CPP studies, GAT211 (20 mg/kg i.p.) and morphine (8 mg/kg i.p.) were combined and dissolved in 20% dimethyl sulfoxide with the remaining 80% consisting of ethanol: emulphor: saline in a 1:1:8 ratio and administered i.p. in a final volume of 10 mL/kg.

### Assessment of Paw Withdrawal Thresholds to Mechanical Stimulation

Paw withdrawal thresholds to mechanical stimulation were measured in grams (g) using an electronic von Frey anesthesiometer (IITC model Alemo 2390–5, Woodland Hills, CA, United States) as described previously (Slivicki et al., 2016). Mice were placed on an elevated metal mesh table where they were habituated under individual, inverted plastic cages for at least 30 min prior to testing. Following the cessation of exploratory behaviors, a force was applied to the midplantar region of the hind paw with a semiflexible tip connected to the anesthesiometer. Mechanical stimulation was terminated when the mouse withdrew its paw from the mesh surface. The threshold for paw withdrawal was determined in duplicate in each paw; responsiveness in each paw was subsequently averaged into a single determination for each animal.

### Assessment of Cold Responsivity

In the same animals used to assess sensitivity to mechanical stimulation, sensitivity to cold stimulation was measuring using the acetone method as described previously (Deng et al., 2014). Animals were tested on the same wire mesh platform used to assess sensitivity to mechanical stimulation (i.e., animals were not moved between tests). In all instances cold responsivity was measured immediately following testing of mechanical responsivity (within ∼30 min). Using the blunt end of a 1 cc syringe, a drop (5–6 μL) of acetone was applied to plantar surface of the hind paw. Acetone was applied three times to each paw and the amount of time the animal exhibited acetone-evoked behaviors (i.e., lifting, biting, or shaking of the stimulated paw) was recorded over 1 min following acetone application. This procedure was performed three times per paw with at least 3-min intervals between successive stimulations. Acetone responsiveness was calculated as the mean of all six acetone stimulations (i.e., both paws included).

### Paclitaxel-Induced Allodynia

Paclitaxel (4 mg/kg i.p.) was administered once daily every other day for a cycle consisting of 4 injections (i.e., on day 0, 2, 4, and 6). Animals were tested for responsivity to mechanical and cold stimulation on days 0, 4, 7, and 15. Assessments of mechanical and cold stimulation were always performed prior to paclitaxel injections whenever injections and behavioral testing were performed on the same day. All other pharmacological manipulations took place beginning on day 16 post-paclitaxel treatment, when behavioral hypersensitivities are stable as reported previously (Deng et al., 2015b; Slivicki et al., 2018b). Our lab and others (Polomano et al., 2001; Siau et al., 2006; Ward et al., 2011; Zheng et al., 2011; Donvito et al., 2016; Curry et al., 2018) have used this model extensively to characterize effects of distinct approaches to manipulate the endocannabinoid signaling system on the development and maintenance of neuropathic nociception. The C57BL/6 mouse line has been reported to not develop hypersensitivity to heat following paclitaxel treatment (Smith et al., 2004) but hypersensitivity develops under other experimental conditions (Braz et al., 2015). Mechanisms underlying the development of paclitaxel-induced neuropathic allodynia remain incompletely understood. Loss of intraepidermal nerve fibers, abnormal mitochondrial function, infiltration of damage-associated molecular patterns and pro-inflammatory cytokine and chemokines within dorsal root ganglia and spinal cord as well as aberrant brain resting state connectivity have all been implicated in contributing to chemotherapy-induced neuropathy produced by paclitaxel (Ferris et al., 2019; Staff et al., 2020).

### Dose-Response and Drug Combination Studies

Dose-response curves assessing suppression of mechanical and cold allodynia induced by GAT211 (Slivicki et al., 2018b) and morphine (Slivicki et al., 2018a) treatments were generated in paclitaxel-treated mice and reported previously. The dose response studies were conducted concurrently, with all testing conducted by the same experimenter (RAS). The experimenter was blinded to the experimental conditions in all studies. Escalating dosing schedules were employed with successive doses administered every 2–3 days. Behavioral assessments were performed 30 min after each injection of drug or vehicle. ED_50_ values were derived for anti-allodynic efficacy to cutaneous stimulation evoked by both mechanical and cold stimulation. In a separate set of paclitaxel-treated mice, 1:1 combination doses of GAT211 + morphine were administered based on these ED_50_ doses in escalating fashion every 2–3 days; this latter combination data set has not been previously reported. ED_50_ values for suppression of paclitaxel-induced mechanical and cold allodynia were generated from the same animals for a given single drug. Separate groups of paclitaxel-treated mice were used to calculate combination ED_50_s (i.e., for the combination of GAT211 + morphine) for suppression of paclitaxel-induced allodynia for each stimulus modality separately (i.e., because ED_50_s differed depending upon the stimulus modality); both mechanical and cold stimulation were nonetheless tested in all animals in the same order using methods identical to those described above. The 1:1 combination doses derived from individual dose-response curves were as follows for each stimulus modality and drug treatment: Mechanical: GAT211 (0.71, 1.42, 2.84, 5.68, 11.35 mg/kg i.p.), morphine (0.42, 0.84, 1.67, 3.34, 6.68 mg/kg i.p.); Cold: GAT211 (0.62, 1.24, 2.48, 4.95, 9.90 mg/kg i.p.), morphine (0.78, 1.56, 3.13, 6.25, 12.5 mg/kg i.p.). Values were converted to % maximal effect using the formula: (Experimental Value – Post-paclitaxel baseline)/(Pre-paclitaxel baseline – Post-paclitaxel baseline) × 100.

### Chronic Treatment Studies in Paclitaxel-Treated Mice

In a separate set of animals, on day 15 post- initial paclitaxel injection, mice were assigned to receive either vehicle, morphine alone (10 mg/kg i.p.) or a sub-threshold dose (i.e., derived from dose-response curves) of GAT211 (5 mg/kg i.p.) in combination with morphine (10 mg/kg i.p.). This dose of morphine was selected as it is a dose known to produce tolerance to anti-allodynic efficacy in paclitaxel-treated mice in our laboratory under analogous conditions (Lin et al., 2018). Mice were injected (i.p.) once daily for 20 consecutive days with the assigned treatment conditions. Mechanical paw withdrawal thresholds and duration of responding to cold stimulation were recorded on days 0, 4, 8, 12, 16, and 20. See Figure 3A for a schematic of the experimental protocol.

**FIGURE 1:**
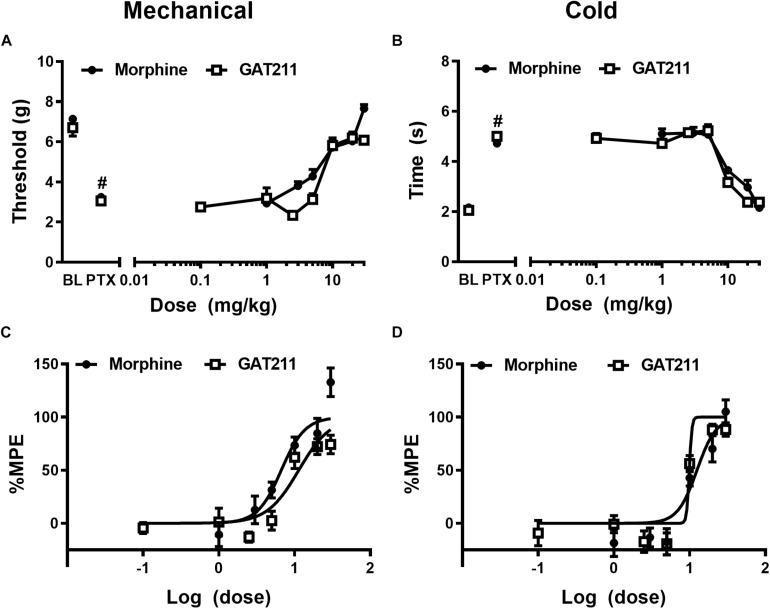
The CB_1_ PAM GAT211 and the opioid analgesic morphine reduce paclitaxel-induced allodynia in a dose-dependent manner. GAT211 (0.1, 1, 2.5, 5, 10, 20, 30 mg/kg i.p.; previously published in Slivicki et al., 2018b) and morphine (1, 3, 5, 10, 20, 30 mg/kg i.p.; previously published in Slivicki et al., 2018a) both dose-dependently reduced paclitaxel-induced behavioral hypersensitivities to **(A)** mechanical and **(B)** cold stimulation. Values were converted to % maximal possible effect (MPE) for **(C)** mechanical and **(D)** cold modalities. Data are expressed as mean ± SEM (*n* = 5–6 per group) *^#^P <* 0.05 vs. pre-paclitaxel values, two-way ANOVA followed by Bonferroni *post hoc* test.

**FIGURE 2:**
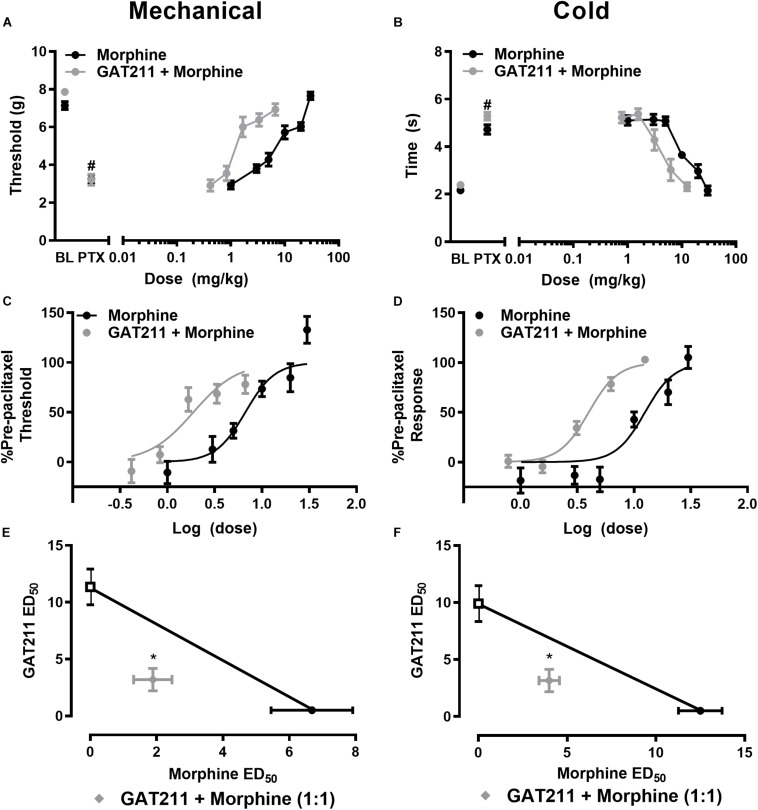
GAT211 synergizes with the opioid analgesic morphine in suppressing paclitaxel-induced allodynia. Co-administration of GAT211 produces a leftward shift in the dose-response curves of morphine to reduce paclitaxel-induced mechanical **(A,C)** and cold **(B,D)** allodynia. Isobolographic analysis revealed a synergistic interaction of GAT211 with morphine in suppressing paclitaxel-induced hypersensitivities to both mechanical **(E)** and cold **(F)** stimulation when administered in a 1:1 ratio. Data are expressed as mean ± SEM (*n* = 6 per group) *P < 0.05 two-tailed *t*-test vs. theoretical additive values. *^#^P <* 0.05 vs. pre-paclitaxel values, two-way ANOVA followed by Bonferroni *post hoc* test.

**FIGURE 3:**
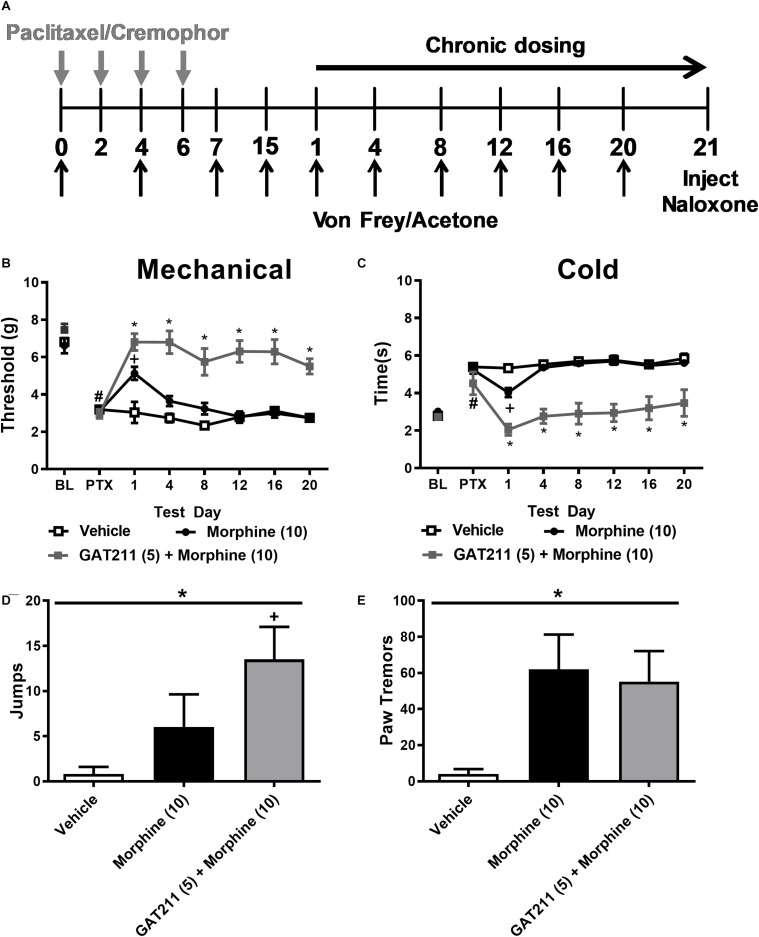
A sub-threshold dose of GAT211 prevents tolerance to the anti-allodynic effects of morphine without exacerbating morphine dependence. **(A)** Schematic shows timing of experimental treatments. The gray vertical arrows show timing of injections (i.p.) of paclitaxel or cremophor-based vehicle. The black vertical arrows show the timing of behavioral testing for assessing responsiveness to mechanical (Von Frey) and cold (acetone) stimulation. The black horizontal arrow shows the duration of once daily chronic dosing. On day 21 naloxone was injected (i.p.) to precipitate opioid withdrawal. GAT211 (5 mg/kg i.p. × 20 days), administered at a sub-threshold dose for reducing paclitaxel-induced allodynia, enhanced efficacy of morphine in reducing hypersensitivities to mechanical **(B)** and cold **(C)** stimulation without the develop of tolerance over a 20-day dosing period. By contrast, tolerance developed to the anti-allodynic efficacy of morphine following repeated dosing. Challenge with naloxone (2 mg/kg i.p.) elicited jumping **(D)** and paw tremor bouts **(E)** in mice treated with morphine (10 mg/kg i.p.) and morphine (10 mg/kg i.p.) co-administered with GAT211 (5 mg/kg i.p.). Co-administration of GAT211 with morphine did not reliably enhance or reduce these behaviors relative to morphine alone. **(B,C)** *^#^P* < 0.05 vs. pre-paclitaxel thresholds, *P < 0.05 GAT211 + Morphine vs. all other groups, ^+^*P* < 0.05 vs. vehicle and GAT211 + morphine (Two-way ANOVA followed by Bonferroni *post hoc* test. Data are expressed as mean ± SEM (*n* = 5–6 per group) **(D,E)** *P < 0.05 overall effect of treatment one-way ANOVA. ^+^*P* < 0.05 one-way ANOVA followed by Bonferroni *post hoc*. Data are expressed mean ± SEM (*n* = 5–6 per group).

### Tail-Flick Antinociception: Dose-Response Analysis

**FIGURE 4:**
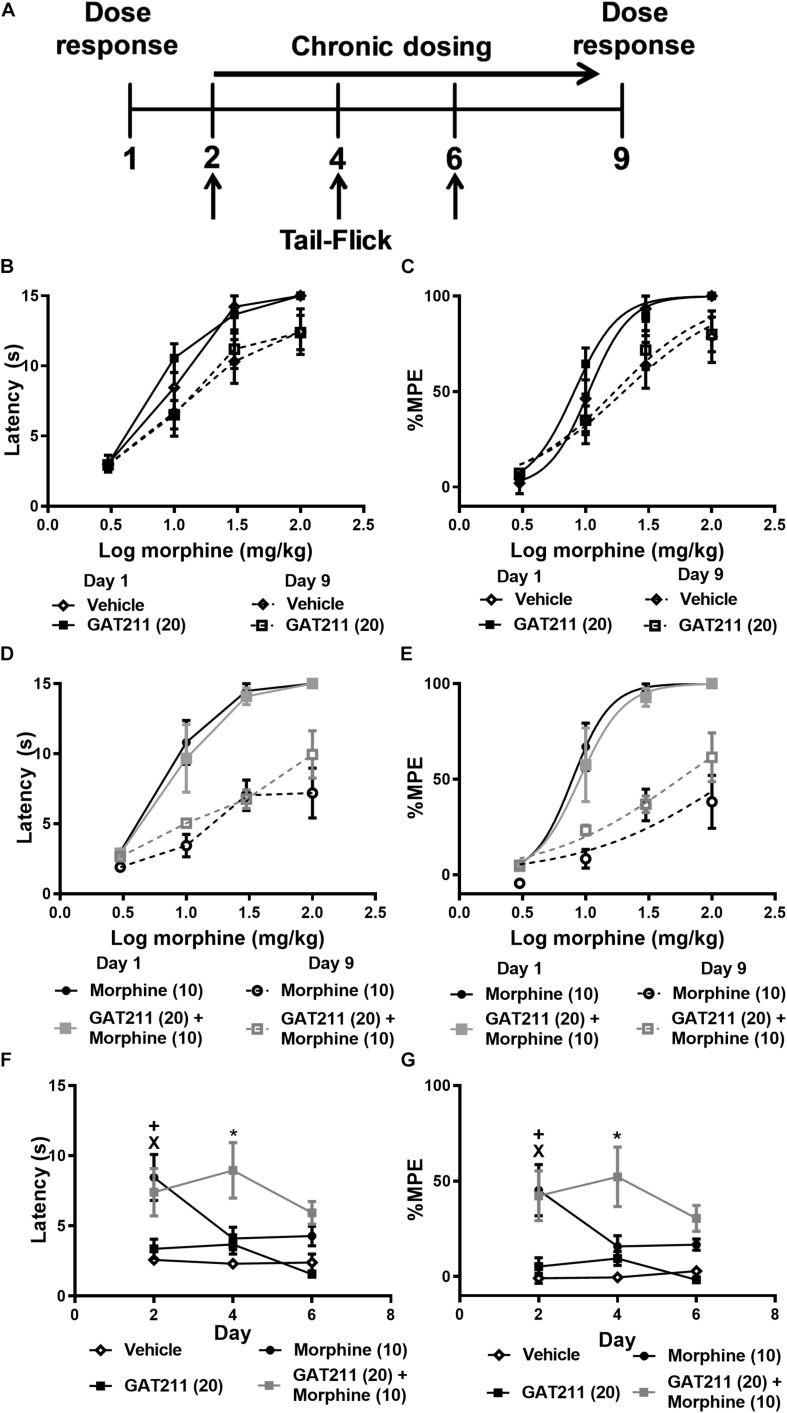
Co-treatment of GAT211 with morphine reduces tolerance to morphine antinociception in the tail-flick test. Schematic shows timing of experimental procedures **(A)**; vertical arrows show time of assessment of tail flick latencies, which were measured 30 min following drug administration (i.p.) on days 2, 4, and 6 of repeated dosing **(A)**. Ascending doses of morphine (0, 1, 3, 10, 30, 100 mg/kg i.p.) produced dose-dependent increases in tail-flick antinociception. Repeated injections of vehicle or GAT211 (20 mg/kg i.p. × 7 days) did not reliably shift the morphine dose response curve **(B,C)**. Repeated injection of morphine (10 mg/kg i.p. × 7 days) produced a right-ward shift in the dose-response curve of morphine in producing antinociception in the tail-immersion test **(D,E)**. The combination of GAT211 (20 mg/kg i.p.) + morphine (10 mg/kg i.p.) also produced a right-ward shift in the dose-response curve for morphine to produce tail-flick antinociception albeit to a lesser degree **(D,E)**. Tolerance to morphine-induced antinociception in the tail immersion test was delayed by co-treatment with GAT211 (20 mg/kg i.p.) **(F,G)**. GAT211+ morphine cotreatment produced heightened antinociception on day 4 but not on day 6 of repeated injections compared to all other groups **(F,G)**. Vehicle and GAT211 do not elicit tail-flick antinociception when administered alone **(F,G)**. Data are expressed as tail-flick latencies in seconds **(B,D,F)** and values transformed to % MPE **(C,E,G)** values. ^X^*P* < 0.05 morphine vs. Vehicle and GAT211, ^+^*P* < 0.05 GAT211 + morphine vs. Vehicle and GAT211 *P < 0.05 GAT211 + morphine vs. all other groups, two-way ANOVA followed by Bonferroni *post-hoc*. Mean ± SEM (*n* = 6 per group).

The hot water tail-immersion test was used to assess the latency to withdraw the tail from a 53–54°C water bath in the absence of paclitaxel treatment. The distal 2 cm of the tail was immersed in the water bath and the latency to elicit a ‘flick’ response was measured as previously described (Bohn et al., 1999; Kim et al., 2015). Prior to injection, three different baseline values were recorded (i.e., with a 10-min interval between successive stimulations). A cut-off of 15 s was applied to avoid tissue damage. A within subjects dose-response curve was calculated using escalating doses of morphine (0, 1, 3, 10, 30, 100 mg/kg i.p.) administered 30 min apart. Approximately 24 h after the last morphine injection, mice were randomized to receive once daily chronic treatments with either vehicle, GAT211 (20 mg/kg i.p.), morphine (10 mg/kg i.p.), or GAT211 (20 mg/kg i.p.) + morphine (10 mg/kg i.p.) for seven consecutive days (days 2–8). Mice were then tested for tail-flick withdrawal latencies 30 min following injection of the aforementioned pharmacological treatments on days 2, 4, and 6 of chronic dosing. On day 9, mice received the same escalating doses of morphine as delivered on day 1. Values were converted to %MPE to compare antinociceptive effects of morphine following acute (i.e., day 1) and chronic (i.e., day 9) drug treatments. See Figure 4A for time course of the experimental protocol.

### Assessment of Naloxone-Precipitated Opioid Withdrawal in Paclitaxel-Treated Mice

On day 21 of chronic drug treatment, mice were treated once again with a terminal injection of their assigned drug condition. Then, 60 min following this injection, the same mice were challenged with naloxone (2 mg/kg i.p.) to precipitate μ opioid receptor-dependent withdrawal behaviors. Mice were video recorded throughout the entire period. The number of naloxone-precipitated jumps and number of bouts of paw tremor behaviors were measured over 30 min following naloxone challenge. All videos were scored by an experimenter blinded to all treatment conditions (SM).

### Assessment of Naloxone-Precipitated Opioid Withdrawal in Otherwise Naïve (i.e., Non-neuropathic) Mice

Mice were anesthetized with isoflurane anesthesia and surgically implanted with a morphine (75 mg) pellet subcutaneously just above the nape of the neck. Subsequently, 71.5 h following pellet implantation, mice were treated acutely with either GAT211 (20 mg/kg i.p.) or an equivalent volume of vehicle. At 72 h post-surgery, mice were challenged with 1 mg/kg i.p. of naloxone to precipitate a μ-opioid receptor-dependent withdrawal syndrome as described previously (Lichtman et al., 2001; Ramesh et al., 2011). The number of jumps and the number of bouts of paw tremor behaviors were evaluated for 30 min following naloxone challenge by a blinded scorer (SM).

### Evaluation of the Impact of GAT211 on Conditioned Place Preference to Morphine

We evaluated whether GAT211 would alter the rewarding effects of morphine using a three-chamber conditioned place preference (CPP) apparatus. Procedures used to assess CPP and/or aversion in an unbiased fashion were identical to those published previously by our laboratory (Slivicki et al., 2018b). In this latter study, we showed that a dose of GAT211 (20 mg/kg i.p.) that suppressed neuropathic and inflammatory pain failed to elicit either reward or aversion (Slivicki et al., 2018b). In brief, the CPP apparatus consisted of two chambers with distinct visual cues (vertical and horizontal black and white stripes) and a center neutral (gray) chamber. The time the animal spent in each chamber was recorded over a 30 min test interval. On days 1 and 2, mice were allowed to freely explore the entire apparatus. On day 3, an initial baseline preference assessment was conducted to confirm that mice did not show any bias for a chamber prior to drug pairings. Animals were excluded from the experiment if they spent more than 1440 s (i.e., 80% of time) or less than 360 s (i.e., 20% of time) in either distinct chamber. On days 4–11, mice received 4 repeated pairings of the assigned drug condition (morphine alone or GAT211 + morphine) on day 4, 6, 8, and 10 and received vehicle in the opposite chamber on day 5, 7, 9, and 11. Treatment assignments were randomized and unbiased. Separate groups of mice received vehicle in both chambers. On day 12, mice were evaluated in the drug free state for the time spent in either chamber to assess the impact of GAT211 on CPP to morphine. A drug chamber preference score (Time in drug chamber post-conditioning minus time in drug chamber pre-conditioning) was calculated to compare different pharmacological treatments between groups.

### Analysis

Non-linear regression analyses were used to generate ED_50_ values with 95% confidence limits. Two-Way ANOVAs were used to analyze drug effects in chronic dosing studies and compare baseline and post-paclitaxel responses. For isobolographic analyses, 1:1 combinations based on the individual ED_50_ values of either morphine or GAT211 in suppressing responding to either mechanical or cold stimulation were generated. Doses of both compounds were administered in combination in an ascending fashion for a given stimulus modality. Combination ED_50_ values were derived from the 1:1 combinations and plotted against the theoretical ED_50_ values. Theoretical ED_50_ values were derived as the expected sum of the two compounds when administered based on their independent ED_50_ values as described previously by our group (Tallarida, 2006; Slivicki et al., 2018b). One-way ANOVA was used to evaluate withdrawal jumps and paw tremors in the case of three group comparisons, whereas two-tailed unpaired *t*-tests using Welch’s correction were used to compare dependent measures in the case of two group comparisons. Two way (2 × 2) ANOVAs followed by Bonferroni *post-hoc* tests were used to compare chamber preference times in CPP studies. Two-tailed *t*-tests using Welch’s correction were performed to compare CPP preference scores in the case of two group comparisons, as appropriate. All data was analyzed using GraphPad Prism version 5.02 (GraphPad Software Inc., La Jolla, CA, United States).

## Results

### General Experimental Results

Paclitaxel produced robust hypersensitivities to mechanical (*F*_1,28_ = 709.0, *p* < 0.0001) and cold (*F*_1,28_ = 1067, *p* < 0.0001) stimulation relative to pre-paclitaxel thresholds prior to pharmacological manipulations (Figures 1A,B). In addition, neither pre- nor post-paclitaxel responsivity to mechanical (*F*_5_,_28_ = 1.405, *p* > 0.25) or cold (*F*_5,28_ = 1.557, *p* > 0.20) stimulation differed between treatment groups and the interaction between treatment and time was not significant for either stimulus modality (mechanical: *F*_5_,_28_ = 1.607, *p* > 0.19; cold: *F*_5,28_ = 1.459, *p* > 0.23).

### Morphine and GAT211 Dose-Dependently Reduce Paclitaxel-Induced Hypersensitivities

Paclitaxel produced hypersensitivities to mechanical (*p* < 0.05 vs. pre-paclitaxel values for all groups: Figure 1A) and cold (*p* < 0.05 vs. pre-paclitaxel values for all groups; Figure 1B) stimulation. GAT211 and morphine dose-dependently reduced paclitaxel-induced hypersensitivities to mechanical stimulation with ED_50_’s of 11.35 (8.657 – 14.880) and 6.682 (4.904 – 9.105) mg/kg i.p. (Figures 1A,C), respectively. GAT211 and morphine also produced dose-dependent reductions in paclitaxel-induced hypersensitivities to cold stimulation with ED_50_’s of 9.904 (9.47–10.33) mg/kg i.p. and 12.50 (9.498 – 16.45) mg/kg i.p. (Figures 1B,D), respectively.

### GAT211 Produces a Left-Ward Shift in the Dose-Response of Morphine Anti-allodynic Efficacy

Co-administration of GAT211 with morphine reduced the ED_50_ of morphine from 6.682 (4.904 – 9.105) mg/kg i.p. to 1.886 (1.337 – 2.660) mg/kg i.p. in suppressing paclitaxel-induced hypersensitivity to mechanical stimulation (Figures 2A,C). Co-administration of GAT211 with morphine also produced a leftward shift in the ED_50_ of morphine in suppressing paclitaxel-induced hypersensitivity to cold stimulation from 12.50 (9.498 – 16.45) mg/kg i.p. to 3.991 (3.470 – 4.590) mg/kg i.p. (Figures 2B,D).

### GAT211 Synergizes With Morphine to Reduce Paclitaxel-Induced Mechanical and Cold Allodynia

GAT211 produced synergistic suppressions of mechanical allodynia when co-administered with morphine (Figure 2E). The observed ED_50_ of GAT211 with morphine [5.02 (3.798 – 6.242) mg/kg i.p.] in suppressing mechanical allodynia was lower (*p* < 0.05, two-tailed *t*-test) than the theoretical additive value [ED_50_: 9.016 (7.298 –10.74) mg/kg i.p.] of the 1:1 ED_50_ combination (Figure 2E).

The combination of GAT211 with morphine similarly produced a synergistic suppression of paclitaxel-induced cold allodynia; the observed ED_50_ of the combination of GAT211 + morphine [7.153 (6.219 – 8.227) mg/kg i.p.] in suppressing paclitaxel-induced cold responsiveness was lower (*p* < 0.05, two-tailed *t*-test) than the theoretical additive value [11.20 (9.529 – 12.87) mg/kg i.p.] of the 1:1 ED_50_ combination (Figure 2F).

### A Behaviorally Inactive Dose of GAT211 Prevents Development of Morphine Tolerance

Paclitaxel produced hypersensitivities to mechanical stimulation (*p* < 0.001 vs. pre-paclitaxel thresholds; Figure 3B). Mechanical paw withdrawal thresholds differed post-injection in groups receiving morphine (10 mg/kg i.p.), the combination of GAT211 (5 mg/kg i.p.) + morphine (10 mg/kg i.p.) or vehicle (*F*_2,14_ = 52.58, *p* < 0.0001), mechanical thresholds changed across injection days (*F*_6,14_ = 8.108, *p* < 0.0001) and the interaction between drug treatment and injection day was significant (*F*_12,14_ = 5.513, *p* < 0.0001) (Figure 3B). *Post hoc* comparisons revealed that morphine suppressed paclitaxel-induced mechanical allodynia on day 1 (*p* < 0.01 vs. vehicle) but was no longer effective by day 4 of repeated dosing (*p* > 0.05 vs. vehicle), suggesting that tolerance had developed to morphine anti-allodynic efficacy (Figure 3B). By contrast, the combination treatment (GAT211 + morphine) reduced paclitaxel-induced mechanical hypersensitivity throughout the entire 20-day dosing period (*p* < 0.05 vs. vehicle at all time points; Figure 3B), and was more effective at reversing mechanical allodynia than morphine alone (*p* < 0.05 vs. morphine at all time points; Figure 3B).

Paclitaxel also produced hypersensitivity to cold stimulation (*p* < 0.001 vs. pre-paclitaxel thresholds; Figure 3C). Cold response times differed between groups treated with morphine (10 mg/kg i.p.), GAT211 (5 mg/kg i.p.) + morphine (10 mg/kg i.p.) or vehicle (*F*_2,14_ = 139.6, *p* < 0.0001), cold responsiveness differed across injection days (*F*_6,14_ = 17.56, *p* < 0.0001), and the interaction between treatment and injection day was significant (*F*_12,14_ = 7.498, *p* < 0.0001) (Figure 3C). *Post hoc* comparisons revealed that morphine reduced paclitaxel-induced cold allodynia on day 1 of repeated dosing (*p* < 0.01 vs. vehicle) but was no longer effective by day 4 of dosing (*p* > 0.05 vs. vehicle), suggesting tolerance had developed to its anti-allodynic efficacy (Figure 3C). By contrast, the combination treatment (GAT211 + morphine) reduced paclitaxel-induced cold hypersensitivity throughout the entire 20-day dosing period (*p* < 0.05 vs. vehicle at all timepoints; Figure 3C) and was more effective at reversing cold allodynia than morphine alone (*p* < 0.01 vs. morphine at all time points; Figure 3C).

### A Sub-Anti-Allodynic Dose of GAT211 Does Not Alter Naloxone-Precipitated Opioid Withdrawal in Paclitaxel-Treated Morphine-Dependent Mice

On day 21, paclitaxel-treated mice injected chronically with vehicle, morphine or GAT211 + morphine as described above were challenged with the μ-opioid receptor antagonist naloxone (2 mg/kg i.p.) to potentially unmask μ-opioid receptor-dependent opioid withdrawal. A one-way ANOVA revealed that drug treatment (*F*_2,14_ = 3.949, *p* = 0.0437) altered the number of naloxone-precipitated jumps. *Post hoc* comparisons revealed that mice treated with GAT211 (5 mg/kg i.p.) + morphine (10 mg/kg i.p.) exhibited more jumps relative to vehicle (*p* < 0.05) whereas the number of jumps did not differ between groups receiving morphine alone or GAT211 + morphine (*p* > 0.17) (Figure 3D).

A one-way ANOVA also revealed that drug treatment altered naloxone-precipitated bouts of paw-tremor behaviors (*F*_2,14_ = 33743, *p* = 0.0499; Figure 3E). *Post hoc* comparisons revealed that the number of paw tremor bouts elicited by naloxone were greater in mice receiving either morphine (10 mg/kg i.p.) alone (*p* = 0.062 vs. vehicle) or GAT211 (5 mg/kg i.p.) + morphine (10 mg/kg i.p.) (*p* = 0.082 vs. vehicle) compared with vehicle (Figure 3E). Co-administration of GAT211 with morphine did not increase the overall number of paw tremors relative to morphine alone (*p* > 0.75; Figure 3E). Thus, co-administration of GAT211 with morphine neither dampened nor exacerbated naloxone-precipitated opioid withdrawal.

### Impact of GAT211 Co-administration on Morphine Antinociception in the Tail-Immersion Test

In otherwise naïve mice, ascending doses of morphine (0, 1, 3, 10, 30, 100 mg/kg i.p.) produced dose-dependent increases in tail-flick antinociception within the same subjects, and this effect did not differ between treatment groups prior to initiation of chronic dosing (Figures 4A–E; see also Table 1).

**TABLE 1:** Impact of pharmacological manipulations (see Figure 4A) on morphine antinociception in the tail-flick test.

**Treatment**	**Day 1**	**Day 9**	**ED_50_ Shift**
Vehicle	10.62 (8.034 – 14.04)	20.15 (11.21 – 36.21)	1.90
Morphine (10)	7.874 (6.273 – 9.884)	141.2 (44.34 – 449.7)*	17.93
GAT211 (20)	8.143 (6.441 – 10.29)	17.09 (11.43 – 25.57)*	2.09
GAT211 (20) + morphine (10)	8.981 (6.524 – 12.36)	55.70 (33.96 – 91.37)*	6.20

*Tail-flick latencies did not differ between groups on day one of cumulative dosing. A right-ward shift in the ED_50_ for producing tail-flick antinociception was observed for animals receiving repeated injections of either morphine (10 mg/kg/day i.p.), GAT211 (20 mg/kg/day i.p) or the combination of morphine (10 mg/kg/day i.p.) with GAT211 (20 mg/kg/day i.p), but not vehicle. The most pronounced shift in the ED_50_ was observed in animals treated with morphine (10 mg/kg i.p.) once daily for 7 days. Animals co-treated with morphine (10 mg/kg i.p.) and GAT211 (20 mg/kg i.p.) displayed a reduced right-ward shift relative to animals treated with morphine alone. Data are expressed as ED_50_ (Confidence Interval, CI), generated from linear regression analysis. ED_50_ doses in mg/kg i.p. with 95% confidence intervals (CI) are shown.*

After 7 subsequent days of once-daily dosing with either vehicle, morphine alone (10 mg/kg i.p.), GAT211 alone (20 mg/kg i.p.) or GAT211 (20 mg/kg i.p.) + morphine (10 mg/kg i.p.), mice were again exposed to the same ascending morphine dose schedule as delivered on day 1 (Figure 4A). Right-ward dose-response shifts were observed in all treatment groups aside from vehicle (Figures 4B–E and Table 1), consistent with the development of tolerance to morphine antinociception. The most pronounced shift was observed in animals treated with morphine (10 mg/kg i.p.) alone; this group displayed a 17.93 fold shift, compared to vehicle-treated animals, which displayed a 1.90 fold shift (Figures 4C,E and Table 1). Co-treatment with GAT211 tended to reduce the overall right-ward shift in morphine antinociceptive efficacy induced by chronic morphine treatment [141.2 (44.34 – 449.7) mg/kg i.p. vs. 55.70 (33.96 – 91.37) mg/kg i.p.] (Figure 4E and Table 1). However, in this latter case, the 95% confidence intervals exhibit some overlap and cannot be deemed significant.

Tail-flick latencies were evaluated on days 2, 4, and 6 of chronic dosing 30 min after the assigned pharmacological treatment. Tail-flick latencies differed between treatment groups when data were represented as either untransformed scores (*F*_3,20_ = 11.43, *p* < 0.0001; Figure 4F) or % MPE (*F*_3,20_ = 15.04, *p* < 0.0001; Figure 4G). Tail-flick latencies on days 2, 4, and 6 differed irrespective of drug treatment only when untransformed scores (i.e., tail-flick latencies in seconds) were analyzed (*F*_2,20_ = 4.343, *p* < 0.028). This difference was not observed when values were converted to %MPE (*F*_2,20_ = 2.72, *p* > 0.11). The interaction between treatment and time was not significant for either tail-flick latencies (*F*_6,20_ = 2.280, *p* > 0.05; Figure 4F) or %MPE (*F*_6,20_ = 1936, *p* > 0.09; Figure 4G). Planned comparisons revealed that on day 2, groups receiving morphine and GAT211+ morphine had higher tail-flick latencies compared to groups receiving either GAT211 or vehicle alone (*p* < 0.05 all comparisons). On day 4, tail-flick antinociception was elevated in the GAT211+ Morphine group relative to all other groups (*p* < 0.05) and by day 6, groups did not differ from each other (Figures 4F,G), consistent with development of antinociceptive tolerance induced by morphine.

### GAT211 Does Not Alter Naloxone-Precipitated Opioid Withdrawal in Morphine-Pelleted Mice

**FIGURE 5:**
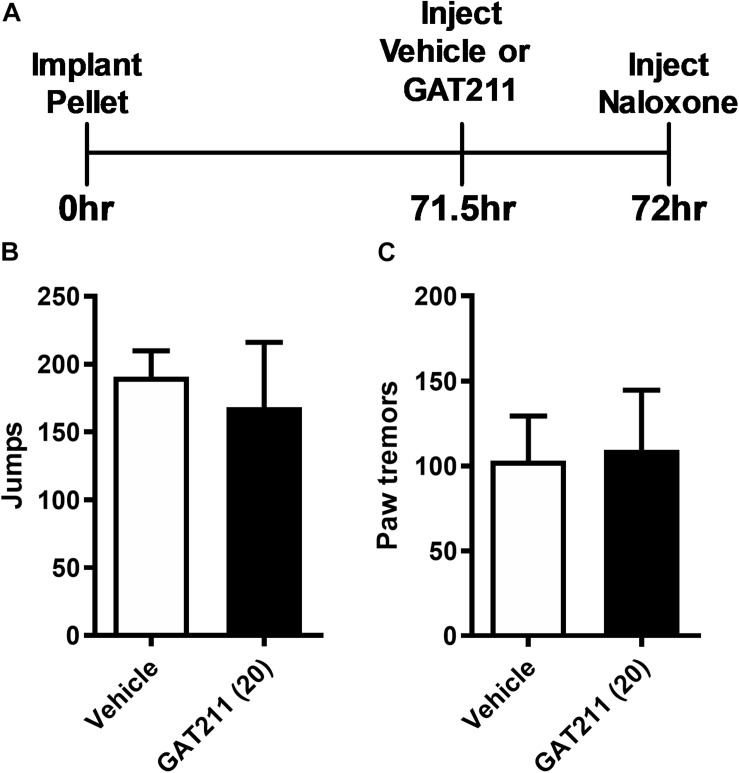
GAT211 does not alter somatic expression of morphine dependence in morphine-pelleted mice. Timeline of the behavioral protocol **(A)**. In mice implanted subcutaneously with a 75 mg morphine pellet, naloxone (1 mg/kg i.p.) challenge did not alter the number of jumps **(B)** or bouts of paw tremors **(C)** following pretreatment with either GAT211 (20 mg/kg i.p.) or vehicle. Naloxone was injected 72 h following morphine pellet implantation. Data are expressed mean ± SEM (*n* = 6 per group).

In morphine-pelleted mice, pretreatment with GAT211 (20 mg/kg i.p.) prior to naloxone (1 mg/kg i.p.) challenge (Figure 5A) did not alter naloxone-precipitated jumping (*t*_6.088_ = 0.4295, *p* = 0.68; Figure 5B) or paw tremor behaviors (*t*_1.755_ = 0.1481, *p* = 0.89; Figure 5C).

### GAT211 Does Not Alter CPP to Morphine

**FIGURE 6:**
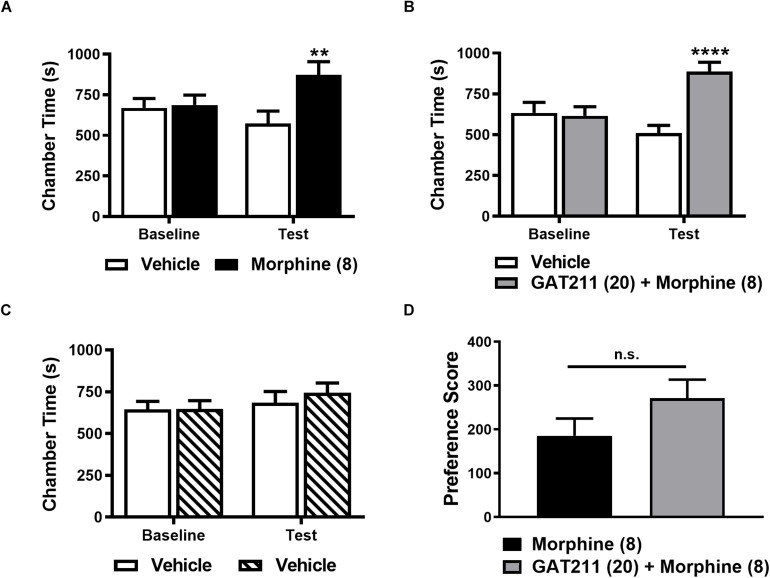
GAT211 does not alter conditioned place preference to morphine. Morphine (8 mg/kg i.p. × 4 pairings) increases the time spent in the drug-paired chamber relative to the vehicle-paired chamber on the test day **(A)**. The combination of GAT211 (20 mg/kg i.p.) and morphine (8 mg/kg i.p.) produces CPP relative to the vehicle-paired chamber on the test day **(B)**. No difference in chamber preference times were observed pre-conditioning (baseline) in any study **(A–C)**. Vehicle-vehicle pairings do not result in preference for any chamber **(C)**. Chamber preference scores did not differ reliably in mice receiving morphine alone or morphine in combination with GAT211 (two-tailed *t*-test using Welch’s correction) **(D)**. Testing for conditioned place preference/aversion was performed on day 12 in a drug-free state. Data are expressed as mean ± SEM (*n* = 10 per group). **P < 0.01, ****P < 0.001 vs. vehicle-paired chamber, two-way ANOVA followed by Bonferroni’s *post hoc* test. n.s., non-significant.

Morphine (8 mg/kg i.p.) produced a robust CPP relative to the vehicle-paired chamber (Figure 6A). A two-way ANOVA revealed no main effect of drug treatment (*F*_1,18_ = 2.944, *p* = 0.1034) or conditioning phase (*F*_1,18_ = 1.773, *p* = 0.1996). However, as expected, the interaction between conditioning phase and drug treatment was significant (*F*_1,18_ = 17.03, *p* = 0.0006; Figure 6A). *Post hoc* comparisons revealed that only morphine-pairing increased time spent in the drug-paired chamber on the test day relative to baseline (*p* < 0.003; Figure 6A), consistent with the development of CPP.

Co-administration of GAT211 (20 mg/kg i.p.) with morphine (8 mg/kg i.p.) similarly produced CPP relative to the vehicle-paired chamber (Figure 6B). Two-way ANOVA revealed that GAT211 + morphine combination treatment altered chamber preference time (*F*_1,18_ = 6.301, *p* = 0.0218) and the interaction between GAT211 + morphine combination treatment and conditioning phase was significant (*F*_1,18_ = 30.93, *p* < 0.0001), whereas conditioning phase (*F*_1,18_ = 4.224, *p* = 0.0547) alone trended to alter chamber preference time. Post-hoc comparisons revealed that animals spent more time in the GAT211 + morphine-paired chamber on the test day, relative to baseline (*p* < 0.001), consistent with development of CPP.

By contrast, time spent in each chamber did not differ when mice received vehicle in both chambers (i.e., vehicle-vehicle pairings; Figure 6C). No main effects of vehicle-vehicle treatment (*F*_1,18_ = 0.2187, *p* = 0.6457) or conditioning phase (*F*_1,18_ = 2.403, *p* = 0.1385) was observed and their interaction (*F*_1,18_ = 0.4332, *p* = 0.5188) was not significant (Figure 6C). Drug chamber preference scores did not differ reliably between groups receiving repeated drug pairings with either morphine alone or GAT211 + morphine (*t*_17.93_ = 1.486, *p* = 0.1548; Figure 6D).

## Discussion

These are the first studies to describe interactions of a CB_1_ PAM with an opioid in models of pain, reward, and physical dependence. Opioid analgesics, while effective, are plagued with a number of adverse side-effects and accounted for approximately 17,500 overdoses in 2015 (National Institute on Drug Abuse, 2012). Cannabis and cannabinoid-based therapies display efficacy in treating a number of different chronic pain states (Hill, 2015; Hill et al., 2017). Although adverse events associated with intake of cannabis or cannabinoids in clinical settings are rare, they produce detrimental side-effects including psychoactivity, tolerance and nausea, among others (Hill, 2015; Hill et al., 2017). In preclinical studies, cannabinoid agonists and inhibitors of endocannabinoid deactivation enhance the antinociceptive effects of opioids while also mitigating unwanted side effects such as tolerance and physical dependence (Cox et al., 2007; Befort, 2015; Wills and Parker, 2016; Wilkerson et al., 2016, 2017). Using a mouse model of chemotherapy-induced peripheral neuropathy, we found that the CB_1_ PAM GAT211 produced synergistic anti-allodynic effects with morphine and prevented development of morphine tolerance. Moreover, GAT211 did not enhance naloxone-precipitated opioid withdrawal, a measure of physical dependence to opioids, in the same subjects relative to morphine treatment alone. Furthermore, GAT211 reduced, but did not fully eliminate, tolerance to morphine antinociception in the absence of neuropathic nociception, as measured by assessments of tail-flick antinociception. Notably, these beneficial effects of GAT211 were observed without an enhancement of opioid-induced reward, as measured by CPP to morphine.

Direct CB_1_ receptor agonists produce antinociceptive efficacy in a number of neuropathic, inflammatory, and visceral preclinical pain models (Rahn and Hohmann, 2009). We previously reported that GAT211, administered alone, decreased paclitaxel-induced hypersensitivities to mechanical and cold stimulation without either the development of tolerance or signs of CB_1_-mediated physical dependence (Slivicki et al., 2018b). The preclinical literature suggests that both direct activation of CB_1_ receptors and inhibitors of endocannabinoid deactivation can enhance morphine’s anti-allodynic effects in models of neuropathic (i.e., chronic constriction injury (CCI) (Kazantzis et al., 2016; Wilkerson et al., 2016, 2017) and chemotherapy-induced toxic neuropathy (Slivicki et al., 2018a), visceral (i.e., acetic acid-induced writhing; Miller et al., 2012) and inflammatory (i.e., CFA-induced inflammatory nociception; Cox et al., 2007) pain. Consequently, we sought to extend these investigations to a CB_1_ PAM. As previously reported, both GAT211 and morphine dose-dependently reduced paclitaxel-induced mechanical and cold allodynia with distinct ED_50_s for each stimulus modality (Slivicki et al., 2018a, b). When administered in a 1:1 combination based on each compound’s ED_50_, synergistic interactions between morphine and GAT211 were observed in reducing paclitaxel-induced mechanical and cold hypersensitivities. In parallel with these findings, GAT211 shifted the dose response curve of morphine leftward for both stimulus modalities, consistent with opioid sparing effects. The mechanism responsible for these synergistic interactions remains to be determined. CB_1_ and μ opioid receptors have similar distribution patterns at a regional, but not necessarily cellular, level. Both receptors are also expressed in the periphery (Richardson et al., 1998; Schmidt et al., 2012), dorsal root ganglia (Hohmann and Herkenham, 1999a, b), dorsal horn of the spinal cord (Hohmann et al., 1999; Salio et al., 2001), as well as areas implicated in the descending control of pain such as the rostral ventromedial medulla and anterior cingulate cortex (Befort, 2015). Thus, the beneficial impact of engaging both CB_1_ and μ opioid receptors in tandem may involve spinal, supraspinal and/or peripheral analgesic mechanisms.

Cannabinoid and opioid systems interact to modulate tail-flick antinociception (Scavone and Van Bockstaele, 2009). Tolerance prevention (Cichewicz and Welch, 2003; Fotio et al., 2020) as well as cross-tolerance between exogenous cannabinoid agonists and morphine have been reported in non-human primates (Gerak et al., 2015) and rats (Altun et al., 2015a, b). In otherwise naïve rats, intra-PAG injection of the cannabinoid receptor agonist HU-210 enhanced morphine-induced antinociception and prevented morphine tolerance in the tail-flick test (Wilson et al., 2008). Low dose combinations of Δ^9^-tetrahydrocannabinol (THC) and morphine retained antinociceptive efficacy without producing downregulation of either μ or CB_1_ receptor protein, as measured by western blot (Cichewicz et al., 2001). Thus, activation of cannabinoid and opioid receptors in tandem, and at a lower than effective dose from either compound administered alone, can produce sustained antinociceptive efficacy without tolerance. The fatty-acid amide hydrolase (FAAH) inhibitors URB597, methyl arachidonyl fluorophosphonate, and the endocannabinoid anandamide have each been reported to increase morphine-induced antinociception (Haller et al., 2008; Pacheco Dda et al., 2009). Thus, we postulated that a CB_1_ PAM theoretically would enhance morphine antinociceptive efficacy and reduce opioid tolerance. In line with our previous findings (Slivicki et al., 2018b), morphine produced a measurable dose-dependent antinociceptive effect in the tail-flick test whereas GAT211 was ineffective. Whereas direct cannabinoid agonists shift morphine’s dose-response curve leftward in the tail-flick test, GAT211 did not enhance morphine-induced tail-flick antinociception on day 2 (i.e., the start of chronic dosing). However, animals were not naïve to morphine on this day, and this prior history of morphine exposure may influence responsivity in this assay. Interestingly, GAT211 delayed, but did not fully prevent, morphine tolerance from developing in our assessment of tail-flick antinociception. GAT211, nonetheless, tended to reduce the overall rightward shift produced by chronic morphine treatment in our dose-response analysis of morphine-induced tail-flick antinociception. These latter experiments were conducted in otherwise normal (i.e., paclitaxel naive) animals, whereas in the experiments discussed previously, mice were rendered neuropathic by paclitaxel treatment. It is possible that μ and CB_1_ receptor expression and function may be altered following the induction of neuropathic nociception (Bushlin et al., 2010), which may change the dynamics and time course of the development of opioid tolerance. The high dose of GAT211 employed in this experiment (20 mg/kg i.p.), was shown previously by our group to be ineffective in producing tail-flick antinociception when administered alone either acutely or chronically (Slivicki et al., 2018b). By contrast, a lower dose (5 mg/kg i.p.) of GAT211 suppressed development of tolerance to anti-allodynic effects of morphine in paclitaxel-treated mice. We previously reported no effect of paclitaxel treatment on tail-flick responses in a similar paradigm (Deng et al., 2015a). It is important to note, however, that different stimulus modalities were tested in naïve (i.e., heat) and paclitaxel-treated (mechanical and cold) mice and could contribute to differences in tolerance development. Differences in endocannabinoid tone in injured and non-injured mice, different duration of chronic dosing and/or distinct mechanisms of tolerance development may be unmasked in assessments of allodynia (i.e., assessed in the presence of neuropathic pain) and tail-flick antinociception (i.e., assessed in the absence of neuropathic pain) (Slivicki et al., 2018b).

Confounding motor effects are unlikely to contribute to interpretation of drug effects in our assessments of tolerance and antinociceptive efficacy. GAT211 (20 mg/kg i.p.) did not produce cardinal signs of CB_1_ activation (i.e., it did not produce catalepsy in the ring test or motor ataxia in the rota-rod test) in our previous studies (Slivicki et al., 2018b). Moreover morphine produces locomotor sensitization rather than sedation in mice (Koek et al., 2012). CB_1_ receptor activation has been shown to exhibit a neuroprotective role in a number of different disease (Kendall and Yudowski, 2016); GAT211 alleviates some behavioral abnormalities in a mouse model of Huntington’s disease, consistent with neuroprotective effects (Laprairie et al., 2019). More work is necessary to determine if the neuroprotective function of a CB_1_ PAM may account for the lack of observable tolerance in paclitaxel-treated mice.

In a CCI model of neuropathic pain, sub-threshold doses of the monoacylglycerol lipase (MGL) inhibitor MJN110 and morphine reduced mechanical allodynia without the development of tolerance (Wilkerson et al., 2016). Similarly, the dual FAAH and MGL inhibitor, SA-57, produced additive effects with morphine and combination doses that were ineffective alone reduced mechanical and heat hypersensitivity in the same model (Wilkerson et al., 2017). Further, a subthreshold dose of THC reduced morphine tolerance in an assay of tail-flick antinociception (Smith et al., 2007). In our study, a sub-threshold dose of GAT211 (5 mg/kg i.p.) prevented the development of morphine tolerance and efficaciously reduced paclitaxel-induced allodynia over a 20-day dosing period. This dose of GAT211 did not suppress paclitaxel-induced allodynia when administered alone (Slivicki et al., 2018b). The dose of morphine employed here also produced tolerance to anti-allodynic efficacy in previous studies from our laboratory using the same model of paclitaxel-induced neuropathic pain (Lin et al., 2018). Endogenous opioids and cannabinoids are potential mediators of observed tolerance effects. In rats, THC administration has previously been reported to release met-enkephalin in the nucleus accumbens (Valverde et al., 2001), increase proenkephalin and proopiomelanocortin mRNA in the hypothalamus (Corchero et al., 1999), and increase expression of prodynorphin and proenkephalin in the spinal cord (Corchero et al., 1997). Both THC and methanandamide, a metabolically stable anandamide analog, increase proenkephalin mRNA in the PAG (Manzanares et al., 1998). Therefore, enhancing CB_1_ receptor activity via a CB_1_ PAM could elicit similar effects to direct CB_1_ receptor agonists in stimulating mobilization of endogenous opioid precursors. More work is necessary to understand how CB_1_ PAMs influence endogenous opioid tone relative to exogenous agonists.

Exogenous cannabinoid agonists such as THC reduce somatic signs of opioid withdrawal (Cichewicz and Welch, 2003). In addition, FAAH and MGL inhibitors reduce both precipitated and spontaneous signs of opioid withdrawal in morphine-pelleted mice in a CB_1_-dependent manner (Ramesh et al., 2011, 2013). In our study, naloxone challenge precipitated somatic signs of opioid withdrawal in mice treated chronically with morphine (10 mg/kg i.p. × 21 days) relative to vehicle, as expected. Notably, naloxone-precipitated jumping and paw tremors were not enhanced in mice receiving GAT211 (5 mg/kg i.p. × 21 days) in combination with morphine (10 mg/kg i.p. × 21 days) compared to morphine (10 mg/kg i.p. × 21 days) alone. Thus, GAT211 reduced tolerance to morphine anti-allodynic efficacy but did not reliably alter naloxone-precipitated opioid withdrawal. In otherwise naïve mice implanted with morphine pellets, a paradigm similar to that used previously (Lichtman et al., 2001; Ramesh et al., 2011), GAT211 (20 mg/kg i.p.) pretreatment did not alter somatic signs of naloxone-precipitated opioid withdrawal. Our findings are especially noteworthy because inhibitors of endocannabinoid deactivation can reduce spontaneous somatic withdrawal behavior in this same paradigm (Ramesh et al., 2011). This tolerance-specific effect could be due changes in endocannabinoid tone in areas that modulate pain such as the periaqueductal gray (PAG), spinal cord, and periphery under conditions in which endocannabinoid signaling in other areas associated with opioid dependence (i.e., locus coeruleus; Scavone et al., 2013) are relatively unaltered. Paclitaxel does not alter levels of anandamide or 2-arachidonoylglycerol in either whole brain samples or lumbar spinal cord (Curry et al., 2018). Nonetheless, changes in endocannabinoid tone could be observed in more discrete neural structures and impact our assessments of opioid dependence.

In our study, GAT211 did not enhance opioid reward, as assessed by CPP to morphine. CB_1_ receptors have been implicated in morphine-induced reward and CB_1_ knockout mice also exhibited decreased CPP to morphine (Martin et al., 2000). We previously reported that GAT211 (20 mg/kg i.p.) does not induce place preference or aversion following repeated pairings (Bushlin et al., 2010; Slivicki et al., 2018b). Using the same paradigm, we showed that the combination of GAT211 and morphine, did not enhance CPP to morphine (8 mg/kg i.p.) relative to morphine alone. Our results align well with recent reports suggesting that MJN110 and morphine co-treatment does not alter morphine drug discrimination. Nonetheless, our studies do not preclude the possibility that a CB_1_ PAM could alter morphine reward under other conditions or that a ceiling effect in morphine reward could mask detection of GAT211-enhancement of reward in our study. Our studies specifically employed paclitaxel-naïve mice so that positive reinforcing effects (i.e., reward) could be assessed without the possible confound of negative reinforcing effects (i.e., removal of an aversive pain state). Interestingly the MGL inhibitor MJN110 induced CPP in paclitaxel-treated but not vehicle-treated mice (Curry et al., 2018), suggesting that a CB_1_ PAM may similarly produce negative reinforcement under similar conditions. More work is necessary to evaluate the rewarding effects of GAT211 in the presence of a pathological pain state.

The present studies contribute to an emerging literature describing therapeutically beneficial effects of positive allosteric modulation of cannabinoid CB_1_ receptor signaling, effects that, in combination with morphine, reverse established neuropathic allodynia, and attenuate the development of morphine tolerance without exacerbating adverse side-effects such as opioid reward or physical dependence. Cannabis has also been reported to enhance the antinociceptive properties of oxycodone, an opioid analgesic, without altering the subjective effects of opioids (i.e., “liking”) (Cooper et al., 2018). It is conceivable that CB_1_ PAMs, by producing synergistic anti-allodynic effects and by preventing development of opioid tolerance, may reduce unwanted side-effects of opioid through opioid sparing effects. Clinical studies are required to determine whether CB_1_ PAMs and opioid-based therapies may be used in tandem to elicit therapeutically beneficial effects with a more circumscribed spectrum of unwanted side effects associated with direct activation of opioid or cannabinoid receptors.

## Data Availability Statement

All datasets generated for this study are included in the article/supplementary material.

## Ethics Statement

The animal study was reviewed and approved by Bloomington Institutional Animal Care and Use Committee.

## Author Contributions

RS, VI, and SM conducted the experiments. RS, VI, SM, JC, and AH analyzed the data. SG and GT synthesized ligands and provided the compounds. RS, VI, JC, and AH designed the experiments. AH oversaw the project. RS wrote the manuscript with VI and AH. All authors read and approved the submitted version of the manuscript.

## Conflict of Interest

The CB1 positive allosteric modulator (GAT211) used here has been covered under the patent application US2017/0197918A1 (to GT). The authors declare that the research was conducted in the absence of any commercial or financial relationships that could be construed as a potential conflict of interest.
